# Isolating the impact of a visual search template’s color and form information on search guidance and verification times

**DOI:** 10.3758/s13414-024-02899-2

**Published:** 2024-05-29

**Authors:** Derrek T. Montalvo, Andrew Rodriguez, Mark W. Becker

**Affiliations:** Department of Psychology, Michigan State University, 316 Physic Rd, East Lansing, MI 48823 USA

**Keywords:** Visual search, Search templates, Visual attention

## Abstract

Visual search can be guided by biasing one’s attention towards features associated with a target. Prior work has shown that high-fidelity, picture-based cues are more beneficial to search than text-based cues. However, typically picture cues provide both detailed form information and color information that is absent from text-based cues. Given that visual resolution deteriorates with eccentricity, it is not clear that high-fidelity form information would benefit guidance to peripheral objects – much of the picture benefit could be due to color information alone. To address this, we conducted a search task with eye-tracking that had four types of cues that comprised a 2 (text/pictorial cue) × 2 (no color/color) design. We hypothesized that color information would be important for efficient search guidance while high-fidelity form information would be important for efficient verification times. In Experiment 1 cues were a colored picture of the target, a gray-scaled picture of the target, a text-based cue that included color (e.g., “blue shoe”), or a text-based cue without color (e.g., “shoe”). Experiment 2 was a replication of Experiment 1, except that the color word in the text-based cue was presented in the precise color that was the dominant color in the target. Our results show that high-fidelity form information is important for efficient verifications times (with color playing less of a role) and color is important for efficient guidance, but form information also benefits guidance. These results suggest that different features of the cue independently contribute to different aspects of the search process.

## Introduction

Visual search, the task of finding objects in unknown locations, is a fundamental and vital process that is utilized daily (Alexander et al, 2019). Yet despite its importance, the features guiding this process are not fully understood. It has been suggested that visual search is guided by the viewer adjusting attentional control settings that serve to bias attention toward features associated with the target (Folk et al, 1992). It is has been further suggested that activating a representation of the search target, or a target template, in working memory plays a key role in these attentional control settings, with attention being automatically biased toward the items matching target templates that are actively maintained in working memory (Desimone & Duncan, 1995; Dowd & Mitroff, 2013; Duncan & Humphreys, 1989; Hout & Goldinger, 2015; Soto et al, 2008). This bias helps guide search by constraining attention to objects that share similar properties with the given target but may also result in the involuntary capture of attention by distractors that share features with the search template (Becker et al., 2010; Sawaki & Luck, 2013; Theeuwes et al., 2010).

Studies investigating the guidance of attention by target templates suggest that high-fidelity, picture-based cues matching the target (e.g., a picture of a target shoe) are more beneficial than text-based cues (e.g., the word “shoe”). For instance, Vickery, King, and Jiang (2005) found that search times were faster when a cue was an identical match to the target than when the cue was the same object presented at a different orientation or size. Further they found that picture cues, even those that differed from the target by orientation or size, were more effective than verbal labels of the target. These findings led the authors to conclude that search templates include detailed visual information. However, all their stimuli were gray-scaled and they did not include eye-tracking, which allows one to separate scanning times from verification times.

Expanding on this work, Malcolm and Henderson (2010) compared search performance for text-based cues and high-fidelity picture cues during search of real-world contexts, and found that high-fidelity picture cues reduced both scanning time (the time from the first saccade to the first fixation on the target) and verification time (the amount of time needed to determine that a fixated object is a target or distractor). These findings suggest that having a high-fidelity picture representation of the search target can benefit both search guidance and decision mechanisms.

Similarly, Maxfield and Zelinsky (2012) compared picture-based cues to text-based cues that varied in terms of their level of categorization – with text-based cues presented at either the superordinate (e.g., dessert), basic (e.g., cake), or subordinate level (e.g., chocolate cake). They found that guidance, measured as the time to first fixate the target, improved as the description became more detailed, with the best guidance being found with picture-based cues and the second best with the subordinate-level text-based cues. For rapid guidance, defined as the proportion of trials where the target was the first item fixated, visual and subordinate text-based cues were both equally effective and more effective than basic or superordinate cues. In addition, they found that verification times had a different pattern; while the picture-based cues were still verified most quickly, the basic-level text cues were second fastest, replicating the basic-level advantage in categorization. While a secondary conclusion of this study was that verification processes might benefit from different cue properties, the take-home message of these studies seems to be that search guidance improves as the fidelity of the representation in working memory increases.

However, it is worth noting that the conclusions of these two studies seem somewhat at odds with some of the earliest work investigating search guidance while tracking eye movements. Williams (1967) had participants search an array of 100 geometric shapes for the shape that had a specific two-digit number embedded in it. The shapes varied in terms of their size, color, and shape with four levels of each variable. To help guide search, text-based cues preceded the presentation of the search array. These cues varied in the amount of information provided – cues provided either no guidance information, a single guidance cue (e.g., either the target’s size, color, or shape), two guidance cues (e.g., both the size and color), or three guidance cues (e.g., the size, color, and shape) of the item containing the target number. The eye-tracking results found that guidance based on color was superior to guidance based on shape or size. More importantly, when the cue contained information about both color and size, color and shape, or color, size, and shape, guidance was similar to guidance based on color alone. Thus, unlike Maxfield and Zelinsky (2012), Williams found that guidance did not improve as more detailed information was provided in the cue, and that color was by far the most effective guidance cue.

If color is as important to guidance as Williams’s results suggest, it may raise concerns about the interpretations of other prior results. For instance, Malcolm and Henderson (2010) compared two types of cues – a text-based cue, which had no information about the specific shape/form of the object and no information about the specific color of the object, and a picture-based cue that had specific information about both the shape/form of the target and its color. Since the picture cue added both specific shape/form information and color information, it is possible that the benefit they observed with a picture cue was completely due to the inclusion of color information. That is, the benefit may not be due to a high-fidelity representation of the object in working memory, but simply because the picture includes color information. A similar argument might be able to explain, at least in part, the findings of Maxfield and Zelinsky (2012). Their observed benefit in search guidance for the subordinate text-based cue, relative to the basic level cue, may have been due to the subordinate cue allowing the participant to infer the target color for some of the cues. For instance, their basic level cue of “ice cream” had the subordinate level cues of “chocolate,” “strawberry,” and “mint chocolate chip.” Arguably the main difference between the basic and subordinate cues is that the latter hints at a color. While the subordinate text-based cues did not lead to as effective guidance as the picture-based cues, this might have been because not all of the subordinate cues had strong color associations. For instance, the “Jacket” basic level cue had the corresponding subordinate cues of “Windbreaker” and “Winter Jacket,” neither of which provides a good clue to the target’s color. In short, the difference in guidance observed with subordinate-level cues may have been primarily due to inferred color information, as the amount of color information in the subordinate-level cues was between the picture and basic-level cues.

In sum, we believe the question is still somewhat open about whether high-fidelity form information provided by a picture-based cue supports search guidance above and beyond the color information typically provided in a picture-based cue. Further, from a theoretical perspective, it is unclear why high-fidelity form information in a cue would be of great benefit to search guidance. Guidance allows for more effective fixations on peripheral objects that are likely targets. Presumably a close match between the mental representation of the search template and the visual representation of an object in a search array would benefit guidance. However, given the steep drop-off of visual resolution with eccentricity, the visual representation of peripheral objects would be degraded rather than representing high-fidelity representation of form. In short, the form information from the high-fidelity cue would not match the visual representation of peripheral items, and thus high-fidelity form information in a cue might not be very helpful to search guidance. However, it is also worth noting, that once an object is fixated, the visual representation of the fixated object would be fairly high-fidelity and thus a good match to a picture-based cue, which may benefit verification times.

Based on this theorizing, our initial hypotheses are that color but not form information may be critical to search guidance, while form information may be beneficial to verification times. To test these hypotheses, rather than have only the two types of cues Malcolm and Henderson used – a full color picture of the target and a verbal description without color information of that target, we had four cues that result from a 2 (high-fidelity form information/no high-fidelity form information) × 2 (color information/grayscale information) factorial design. This design, coupled with eye-tracking, allowed us to examine the independent contributions of high-fidelity form information and color information on search guidance and verification times in a search task. In Experiment 1, cues were high-fidelity cues with color (a colored picture of the target), high-fidelity cues without color (a gray-scaled picture of the target), text-based cues that included color (e.g., “blue shoe”), or text-based cues without color (e.g., “shoe”). Experiment 2 was a replication of Experiment 1, except that the color word in the text-based cue with color was presented in the actual color of the target (e.g., “blue shoe”).

Finally, we note that both Malcolm and Henderson (2010) and Maxfield and Zelinsky (2012) attempted to evaluate the effects of cue specificity on both search guidance and verification times. To evaluate verification times, both papers used the time from the first fixation on the target to the execution of the button press indicating that the target was found. We also used this metric as a measure of target verification time. To evaluate search guidance, both papers indexed the time from array onset to the first fixation on the target, and we also used this as a metric of search guidance. While those dependent variables were consistent across those two papers, they each also had some unique variables. Maxfield and Zelinsky (2012) included a rapid guidance variable, which indexed the proportion of trials in which the target was the first object fixated. Malcolm and Henderson (2010) reasoned that there were two processes that occurred during the search process – a process that evaluates whether the currently fixated item is a target or distractor and, if the object is determined to be a distractor, a process that decides which item to fixate next. To differentiate between these two processes, they had two additional metrics. The first was the fixation time on distractors as an index of the process of evaluating individual elements, while the second was the number of scene regions visited during search as an index of guidance. We see value in each of the metrics appearing in both papers and as such have included analyses of all of these variables.

## Method

### Participants

A power analysis (Faul et al., 2007) to detect an effect size of .25 with power of .95 suggested that a sample size of 36 participants would be sufficient.^1^ To allow for attrition of low-performing participants we ran 39 undergraduate students, who participated in the experiment for course credit. Participants were 18–22 years old and had normal or corrected-to-normal color vision.

### Materials

A chin rest placed 57.2 cm from a 20-in. LCD screen was used to stabilize the head while eye movements were tracked by an EyeLink 1000. The experiment was created using Experiment Builder and responses were entered using a Microsoft SideWinder game controller.

### Design

**Fig. 1:**
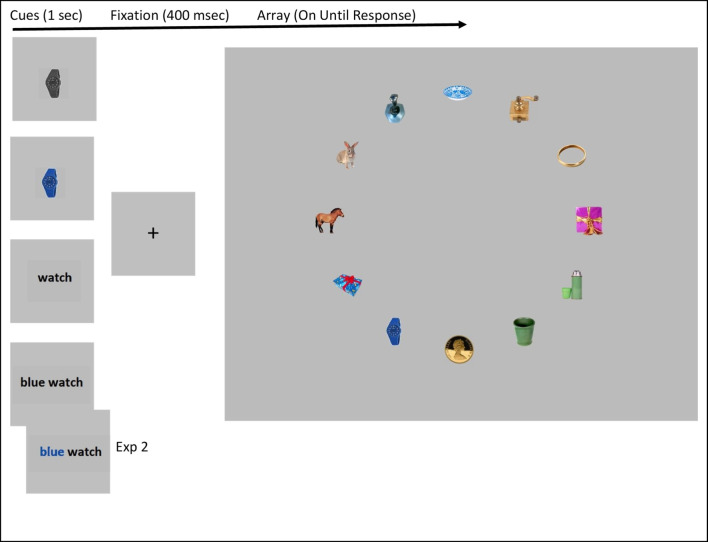
Schematic of the design and stimuli. Note the lowest cue stimulus was the color text-based cue in Experiment 2

The experiment consisted of a total of 184 visual search trials that were broken into four blocks of 46 trials. In each trial the target cue was presented for 1,000 ms, followed by the presentation of a fixation point for 400 ms, which was then followed by a display of 12 color photo-realistic items displayed around an imaginary clock face with a radius of 7.94 degrees of visual angle (see Fig. 1). The search array remained on the screen until a response was indicated by a button press. Each search target was a unique item that appeared only once for each participant, but the identity of each target was counterbalanced such that each target appeared equally often in all experimental conditions across a set of four participants. Each block consisted of a different cuing condition of the two (color/grayscale) × two (picture/text) design. For the text with a color cue condition, the color word was a basic color name (e.g., blue, green, red, yellow, orange, pink, or purple). Within each block one-half of the trials were target-present trials and half were target-absent trials. Block order was randomized across subjects, and trial order within blocks was also randomized.

### Stimuli

Stimuli were selected from the Hemera Photo Objects Volume II (Hemera Technologies, 2001).^2^ Stimuli were scaled so that their longer dimension (height or width) was 60 pixels (~1.7°). To create displays, we first identified a set of 92 unique images that were our target-present cues and a second set of 92 unique images that would serve as the target-absent cues. For each of these 184 images, we created the four types of cues that our design required. We then made four lists, each consisting of 23 target-present and 23 target-absent cues. For a given subject, each of the four lists was assigned to different cueing blocks, so that each subject saw each of the 92 target-present and each of the 92 target-absent cue images only once. Finally, we made four versions of the experiment so that for every four subjects, each target appeared once in each cueing condition. In addition to these target-present and target-absent cue images, we identified an additional 188 unique images that served as distractors in the array (see Fig. 1).

### Procedure

Participants entered a sound-proof booth and were calibrated on the EyeLink after giving informed written consent. Participants were instructed to search for the image described by the cue and were asked to make a target-present/absent decision as quickly and accurately as possible. Auditory feedback was given for incorrect responses. Most participants completed the experiment in less than 45 min.

### Dependent variables

Button press responses were used to measure overall reaction time (RT) and accuracy. Eye-movement recordings were used to divide the overall RT into a scanning and verification phase. To do so we followed the approaches of both Malcolm and Henderson (2010) and Maxfield and Zelinsky (2012). Like both of those papers, we defined verification times as the time from the first fixation on the target to the execution of the button press indicating that the target was found. To evaluate search guidance, both papers indexed the time from array onset to the first fixation on the target, and we also used this as a metric of search guidance.^3^ While those dependent variables were consistent across those two papers, they each also had some unique variables. Maxfield and Zelinsky (2012) included a rapid guidance variable, which indexed the proportion of trials in which the target was the first object fixated. Malcolm and Henderson (2010) had two additional metrics. The first was designed to evaluate the decision mechanism used to determine whether a fixated item was a distractor. To evaluate this decision stage, they used fixation duration on distractors. We also adopted this approach and to eliminate the possible influence of detecting the target on this metric, it was calculated only on the target-absent trials. Their second metric was the number of scene regions visited during search, which they used as an index of guidance. Given that they had real-world scenes, they segmented the scene into 48 equal-sized regions to perform their number-of-regions analysis. Here we have arrays of objects, so the equivalent approach we used to index guidance was to analyze the total number of objects fixated on correct target- present trials.

### Analysis

We eliminated the data from two participants whose accuracy was greater than three standard deviations below the group mean. Data from the remaining 37 participants were analyzed using a series of 2 (cue format: picture/text) × 2 (cue color: color/grayscale) repeated-measures ANOVAs, with separate analyses for each dependent variable. Where appropriate, follow-up paired t-tests were performed to determine the source of the effects. We eliminated trials with extreme RTs, defined as < 100 or > 5,000 ms (Vickery, King & Jiang, 2005), resulting in the elimination of 2.17% of trials.

### Accuracy

#### *Hits*

**Fig. 2:**
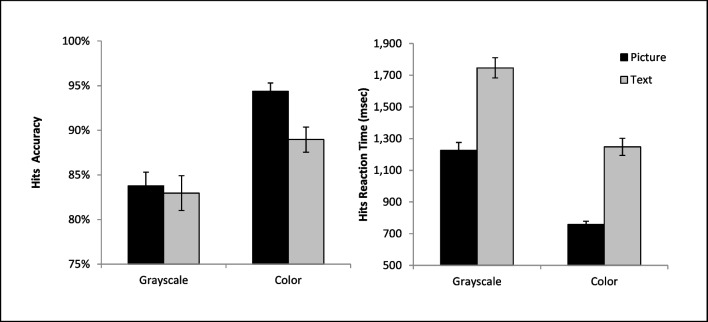
Accuracy rates and mean reaction times are presented for correct target detections (Hits) as a function of condition. Error bars represent the standard error of the mean

The ANOVA on hits revealed a significant main effect of cue format, *F*(1, 36) = 4.43, *p* = .042, η_*p*_^*2*^ = .11, with higher hit accuracy with a picture than text cue. There was also a significant main effect of cue color on accuracy,* F*(1, 36) = 46.54, *p* < .001, η_*p*_^*2*^ = .57, with a higher hit accuracy on colored than gray-scaled cues. These main effects were qualified by a significant interaction, *F*(1, 36) = 4.94, *p* = .033, η_*p*_^*2*^ = .12. The source of this interaction was that the addition of color to the cue had a more pronounced effect on picture than a text-based cue (Fig. 2). In fact, follow-up paired t-tests revealed that the picture cue (*M* = 83.78%, *SD* = 9.32%) and text cue (*M* = 82.96%, *SD* = 11.75%) did not significantly differ from each other when they were gray-scaled, *t*(36) = .40, *p* = .694. However, accuracy was significantly higher for the colored picture cue (*M* = 94.36%, *SD* = 5.69%) than the colored text cue (*M* = 88.95%, *SD* = 8.55%), *t*(36) = 3.65, *p* < .001, and both colored cues produced more hits than the gray-scaled cues, all *p* < .006.

#### *False alarms*

The ANOVA on false alarms revealed a significant main effect of cue format, *F*(1, 36) = 22.69, *p* < .001, η_*p*_^*2*^ = .39, displaying that false alarms occurred less frequently with the picture cue (*M* = 2.59% , *SD* = 3.71%) than the text cue (*M* = 9.40%, *SD* = 8.03%). There was also a significant main effect of cue color, *F*(1, 36) = 2.95, *p* = .094, η_*p*_^*2*^ = .08, with fewer false alarms with a colored (*M* = 5.06%, *SD* = 4.81%) than a gray-scaled (*M* = 6.93%, *SD* = 6.27%) cue. There was no significant interaction found between cue type and cue color, *F*(1, 36) = .22, *p* = .642, η_*p*_^*2*^ = .006.

### Reaction time

The ANOVA on RT (see Fig. 2) for hits revealed a significant main effect of cue format, *F*(1, 36) = 206.41, *p* < .001, η_*p*_^*2*^ = .85, with faster RTs with a picture than a text cue. There was also a significant main effect of cue color, *F*(1, 36) = 185.98, *p* < .001, η_*p*_^*2*^ = .84, with faster RTs for the colored cue compared to the gray-scaled cue. No significant interaction was found between factors, *F*(1, 36) = .22, *p* = .64, η_*p*_^*2*^ = .006.

Following Vickery et al. (2005) and Maxfield and Zelinsky (2012), we did not analyze the correct rejection trials – the decision to quit a search trial is complex and makes interpretation of these target-absent RTs difficult (Chun & Wolfe, 1996).

### Scanning phase

**Fig. 3:**
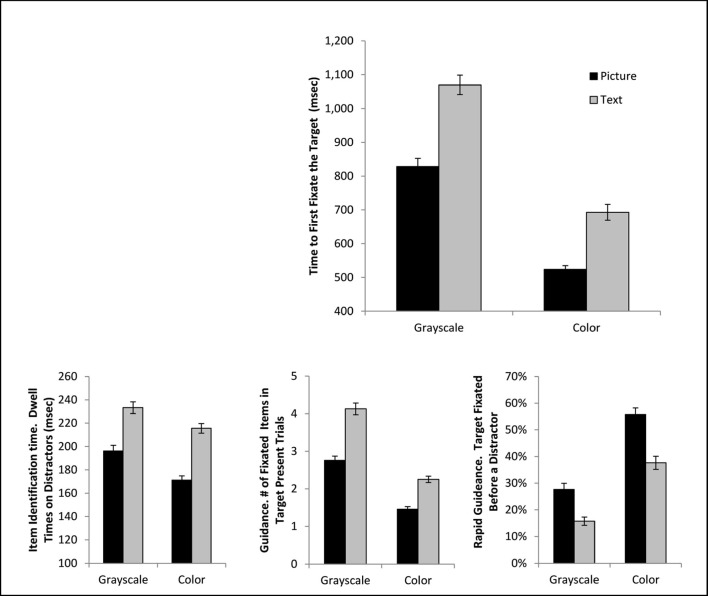
The top panel presents mean total scanning time (the time from array onset to the first fixation on the target) as a function of condition. In the bottom row, the lower left and middle panels present the subcomponents of the scanning time, namely the time required to identify a fixated item as a distractor and the number of items fixated during a trial. The bottom right depicts rapid guidance (the percentage of trials where the target was fixated before another array item). Error bars depict standard error of the mean

The overall scanning phase was defined as the time from array onset to the start of the first fixation that was on the target. The ANOVA on the scanning phase revealed a significant main effect of cue format, *F*(1, 36) = 97.49, *p* < .001, η_*p*_^*2*^ = .73, cue color, *F*(1, 36) = 347.38, *p* < .001, η_*p*_^*2*^ = .91, and a significant interaction, *F*(1, 36) = 5.54, *p* = .024, η_*p*_^*2*^ = .13 (Fig. 3). This pattern emerged because adding both picture information and color information to the cue reduced the time required to complete the scanning phase; however, the addition of color information produced a larger benefit for the text-based than the picture cues. For scanning duration, the addition of color seems to have a major impact, with the colored-text cue producing briefer scanning phases than the gray-scaled picture cue, *t*(36) = 5.38, *p* < .001, *d* = .89.

As mentioned above, one factor that can influence the overall scanning phase duration is the amount of time it takes to identify that the currently fixated item is a distractor. An ANOVA on distractor fixation times during target-absent trials revealed a significant main effect of cue format, *F*(1, 36) = 195.08, *p* < .001, η_*p*_^*2*^ = .84, and cue color, *F*(1, 36) = 36.19, *p* < .001, η_*p*_^*2*^ =.50. The two factors did not interact, *F*(1, 36) = 2.23 *p* = .14, η_*p*_^*2*^ = .058. Having a picture cue resulted in dramatically faster distractor identification times. While adding color also reduced identification times, the influence was somewhat less pronounced, such that a grayscale picture cue produced briefer distractor fixations than a colored text-based cue, *t*(36) = 4.78, *p* < .001, *d* = .79.

A second factor contributing to the overall scanning duration is the efficiency of search guidance when selecting items to fixate. Following Malcolm and Henderson (2010), the number of unique items fixated in a target-present trial provides an index of the efficiency of search guidance. An ANOVA on this variable revealed a significant main effect of cue format, *F*(1, 36) = 151.70, *p* < .001, η_*p*_^*2*^ = .81, cue color, *F*(1,36) = 265.60, *p* < .001, η_*p*_^*2*^ = .88, and a significant interaction, *F*(1, 36) = 12.09, *p* = .001, η_*p*_^*2*^ = .25. Again, both picture information and color information were beneficial to guidance, but the color information was particularly helpful for the text-based cue. Again, a text-based color cue resulted in better search guidance than a gray-scaled picture cue, *t*(36) = 5.13, *p* < .001, *d* = .84.

Following Maxfield and Zelinsky (2012), we also investigated the efficiency of cues for rapid guidance, defined as the proportion of target-present trials where the target item was the first item fixated in an array. An ANOVA on this variable revealed a significant main effect of cue format, *F*(1, 36) = 66.15, *p* < .001, η_*p*_^*2*^ = .65, cue color, *F*(1, 36) = 168.94, *p* < .001, η_*p*_^*2*^ = .82, and no significant interaction, *F*(1, 36) = 2.79, *p* = .10, η_*p*_^*2*^ = .072. Again, although both color and picture information increased rapid guidance, the impact of color was rather large, such that a colored text cue led to better rapid guidance than a gray-scaled picture cue, *t*(36) = 3.61, *p* < .001, *d* = .59.

### Verification times

**Fig. 4:**
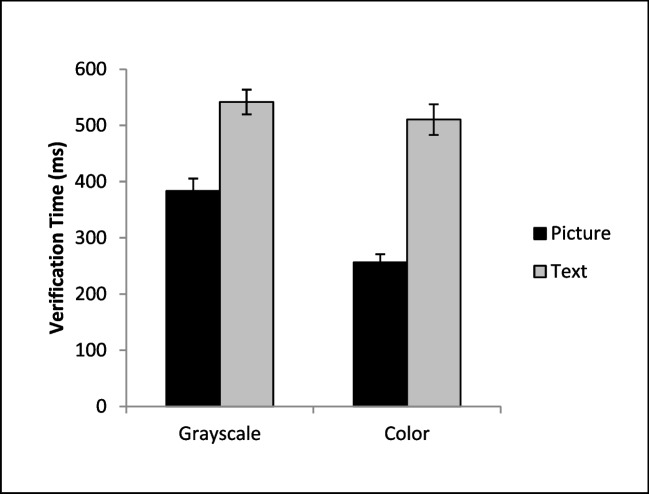
Mean verification times as a function of condition. Error bars depict the standard error of the mean

The time required to execute a button press after reaching the target is another factor that contributes to the overall RT for the task. An ANOVA on verification times (Fig. 4) revealed a significant main effect of cue format, *F*(1, 36) = 235.66, *p* < .001, η_*p*_^*2*^ = .87, cue color, *F*(1, 36) = 37.60, *p* < .001, η_*p*_^*2*^ = .51, and a significant interaction, *F*(1, 36) = 8.63, *p* = .006, η_*p*_^*2*^ = .19. The source of this interaction was due to color significantly reducing verification times for the picture cues, *t*(36) = 6.84, *p* <.001, *d* = 1.12, but there was no benefit of adding color to the text-based cue, *t*(36) = 1.37, *p* = .09, *d* = .18

## Discussion

The results from Experiment 1 support a number of conclusions. First, verification times were much faster for picture- than text-based cues. This pattern is in line with our hypothesis that a high-fidelity representation should be of benefit to the verification process because the target is at fixation, and thus the visual representation should also be high-fidelity. In addition, adding color to a picture cue results in extremely fast verification times. These very fast verification times likely result because the addition of color to a picture cue results in a perfect match between the cue and target. However, it is notable that adding color information to the text-based cue did not produce faster verification times. The lack of a color benefit for the text-based cue suggests that having the abstract color label and the visual input match is necessary but not sufficient for verification. That is, when the color information between a cue and target matches, one must still determine whether the form of the object is consistent with the verbal description of the target.

In this experiment, adding color information to a text-based cue reduced dwell times on distractors, suggesting that when abstract color information in a cue is a mismatch with a currently fixated object, the mismatch is sufficient for determining that object is not the target. However, to preview, this result did not replicate in Experiment 2, so we do not put much emphasis on this result.

Experiment 1 also suggests that color plays a major role in improving guidance. For all dependent variables associated with the efficient selection of items to fixate during search (scanning time, rapid guidance, and the number of fixated items), a text-based cue including information about the color of the target outperformed picture-based cues devoid of color. These findings are generally consistent with prior work suggesting that color information is dominant when features are being used to constrain attention to likely targets (Williams, 1967). It is also consistent with our hypothesis that much of the previously reported guidance benefit of picture-based over text-based cues may be due to the inclusion of color information in the cue rather than having a high-fidelity representation.

However, it is worth noting that the guidance for a text-based cue with color information was not as strong as guidance for a colored picture-based cue. Thus, the results from Experiment 1 do not support our extreme supposition that all of the guidance benefit of picture-based over text-based cues might be due to the inclusion of color information. However, it is possible that our extreme supposition is correct, but we failed to find support for it in Experiment 1 because the specificity of the color information was higher for colored picture cues than for colored text cues. In the color picture-based cue, the precise color of the cue was identical to the color of the target. By contrast, in the color text-based cue condition, the cue indicated a color category, but not a specific value within that category. This difference in color specificity may have resulted in significantly better guidance for the colored picture cue. To investigate this possibility, in Experiment 2 we added the precise color of the dominant color of the target to the text-based color cue.

## Experiment 2

The goal of Experiment 2 was to perform a replication while adding precise color information about the target color into the text-based cue. To do so, we used the eye-dropper tool in Adobe Photoshop, to identify the dominant color of the targets, and then presented the color word in the colored text-based cue in that precise color. For instance, rather than the cue being “blue watch,” it became “blue watch” with the color for blue matching the dominant color in the target image (Fig. 1). In all other respects, the methods of Experiment 2 were identical to Experiment 1.

### Analysis

Forty-three undergraduate students participated in the experiment for course credit. We eliminated the data from two participants whose accuracy was greater than three standard deviations below the group mean. Data from the remaining 41 participants were analyzed using a series of 2 (cue format: picture/text) × 2 (cue color: color/grayscale) repeated-measures ANOVAs, with separate analyses for each dependent variable. Where appropriate, follow-up paired t-tests were performed to determine the source of the effects. For the RT analyses, again we eliminated trials with extreme RTs < 50 or > 5,000 ms, resulting in the elimination of 1.6% of trials.

### Accuracy

#### *Hits*

**Fig. 5:**
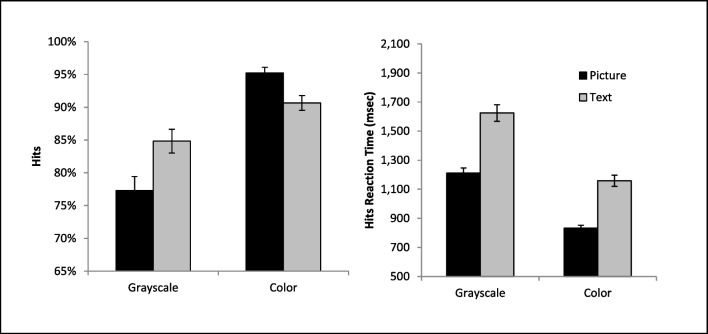
Accuracy rates and mean reaction times for Experiment 2 are presented for correct target detections (Hits) as a function of condition. Error bars represent the standard error of the mean

The ANOVA on hits revealed a significant main effect of cue color,* F*(1, 40) = 62.74, *p* < .001, *η*_*p*_^*2*^ = .61, with higher hit accuracy found with colored than gray-scaled cues. The main effect of cue format was not significant, *F*(1, 40) = 1.59, *p* = .22, *η*_*p*_^*2*^ = .038. However, this was qualified by a significant interaction, *F*(1, 40) = 22.84, *p* < .001, *η*_*p*_^*2*^ = .36. The source of this interaction was that the text-based cue produced higher accuracy than the picture-based cue, *t*(40) = 3.59, *p* < .001, *d* = .56, when they appeared in grayscale (Fig. 5). However, when they appeared in color, this pattern reversed, with higher accuracy for picture-based cues, *t*(40) = 3.64, *p* < .001, *d* = .57. We believe that the low performance with the gray-scaled picture cue might be caused by subjects having difficulty identifying the pictures when they were gray-scaled (Bramão et al., 2011). Even so, the low hit performance for the gray-scaled picture cues means that the faster RTs for that condition (see below) could be due to a speed-accuracy tradeoff. While not ideal, this possibility does not overly concern us given that the metrics below for search guidance suggest that there is good guidance with the gray-scaled cues.

#### *False alarms*

The ANOVA on false alarms revealed a significant main effect of cue format, *F*(1, 40) = 25.99, *p* < .001, *η*_*p*_^*2*^ = .39, with fewer false alarms for picture- (*M* = 1.96%, *SD* = 2.31%) than text-based (*M* = 10.34%, *SD* = 10.82%) cues. There was also a significant main effect of cue color, *F*(1, 40) = 1.22, *p* = 0.277, *η*_*p*_^*2*^ = .03, with fewer false alarms with colored (*M* = 5.78%, *SD* = 6.85%) than gray-scaled (*M* = 6.52%, *SD* = 5.38%) cues. There was no significant interaction found between these factors, *F*(1, 40) < .001, *p* >.99, *η*_*p*_^*2*^ < .001.

### Reaction time

The ANOVA on target-present RTs revealed a significant main effect of cue format, *F*(1, 40) = 126.78, *p* < .001, *η*_*p*_^*2*^ = .76, with faster RTs observed with picture than text-based cues. There was also a significant main effect of cue color on RTs, *F*(1, 40) = 249.85, *p* < .001, *η*_*p*_^*2*^ = .86, with faster RTs with colored than gray-scaled cues. There was also a significant interaction, *F*(1 ,40) = 7.02, *p* = .011, *η*_*p*_^*2*^ = .15 (Fig. 5). The source of this interaction seems to be that the reduction in RT caused by the addition of color to the cue was more pronounced for text (*M* = 466.25, *SE* = 34.44) than picture-based cues (*M* = 379.34, *SE* = 27.96).

### Scanning phase

**Fig. 6:**
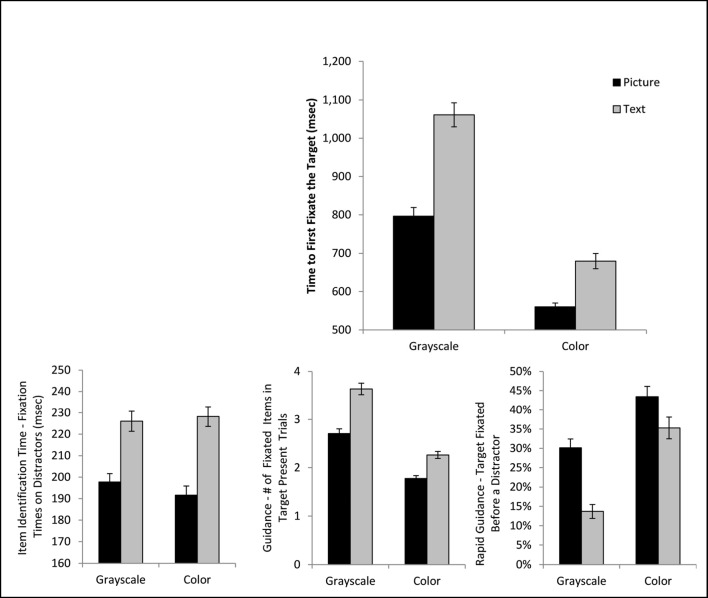
The top panel presents mean total scanning time as a function of condition for Experiment 2. In the bottom row, the lower left and middle panels present the subcomponents of the scanning time, namely the time required to identify a fixated item as a distractor and the number of items fixated during a trial. The bottom right depicts rapid guidance (the percentage of trials where the target was fixated before another array item). Error bars depict standard error of the mean

The time from array onset to the first fixation on the target was defined as the overall scanning phase. The ANOVA on the scanning phase revealed a significant main effect of cue format, *F*(1, 40) = 86.36, *p* < .001, η_*p*_^*2*^ = .68, cue color, *F*(1, 40) = 272.24, *p* < .001, η_*p*_^*2*^ = .87, and a significant interaction, *F*(1, 40) = 23.59, *p* < .001, η_*p*_^*2*^ = .37 (Fig. 6). Similar to Experiment 1, this pattern emerged because adding both picture and color information to the cue reduced the time required to complete the scanning phase; however, the addition of color information produced a larger benefit for the text- than the picture-based cues. Indeed, for scanning duration the addition of color seemed to have a major impact, with a colored-text cue producing briefer scanning phases than a gray-scaled picture cue, *t*(40) = 4.74, *p* < .001, *d* = .74.

As mentioned above, one factor that can influence the overall scanning phase duration is the amount of time it takes to identify that the currently fixated item is a distractor. An ANOVA on distractor fixation times during target-absent trials revealed a significant main effect of cue format, *F*(1, 40) = 204.53, *p* < .001, η_*p*_^*2*^ = .84. Unlike Experiment 1, the main effect of cue color, *F*(1, 40) = 1.32, *p* = .26, η_*p*_^*2*^ = .032, did not reach significance, and these two factors did not interact, *F*(1, 40) = 3.80, *p* = .58, η_*p*_^*2*^ = .087. Having a picture-based cue resulted in dramatically faster distractor identification times. However, unlike Experiment 1, adding color did not provide an additional benefit, an issue we further address in the discussion.

A second factor contributing to the overall scanning duration is the efficiency of search guidance when selecting items to fixate. Following Malcolm and Henderson (2010), the number of items fixated in a target-present trial provides an index of the efficiency of search guidance. An ANOVA on this variable revealed a significant main effect of cue format, *F*(1, 40) = 127.26, *p* < .001, η_*p*_^*2*^ = .76, cue color, *F*(1, 40) = 269.87, *p* < .001, η_*p*_^*2*^ = .87, and a significant interaction, *F*(1, 40) = 16.39, *p* <.001, η_*p*_^*2*^ = .29 (Fig. 6). Again, both picture information and color information were beneficial to guidance, but the color information was particularly helpful for the text-based cue as text-based color cues resulted in better search guidance than gray-scaled picture-based cues, *t*(40) = 5.12, *p* < .001, *d* = .80.

Following Maxfield and Zelinsky (2012), we also investigated the efficiency of cues for rapid guidance, which is defined as the proportion of target-present trials where the target item was the first item fixated in an array. An ANOVA on this variable revealed a significant main effect of cue format, *F*(1, 40) = 43.83 *p* < .001, η_*p*_^*2*^ = .52, cue color, *F*(1, 40) = 86.16, *p* < .001, η_*p*_^*2*^ = .68, and a significant interaction, *F*(1, 40) = 5.03, *p* =.03, η_*p*_^*2*^ = .11 (Fig. 6). Again, although both color and picture information increased rapid guidance, the impact of color was more pronounced for text-based cues, such that a colored text cue led to marginally better rapid guidance than a gray-scaled picture cue, *t*(40) = 1.95, *p* = .06, *d* = .30.

### Verification times

**Fig. 7:**
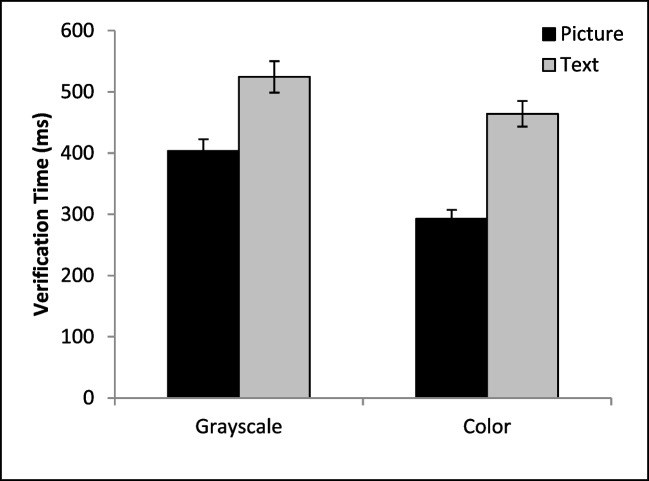
Mean verification times as a function of condition. Error bars depict the standard error of the mean

The time required to execute a button press after reaching the target is another factor that contributes to the overall RT for the task. An ANOVA on verification times revealed a significant main effect of cue format, *F*(1, 40) = 134.14, *p* < .001, η_*p*_^*2*^ = .77, cue color, *F*(1, 40) = 52.27, *p* < .001, η_*p*_^*2*^ = .57, and a significant interaction, *F*(1, 40) = 8.357, *p* = .006, η_*p*_^*2*^ = .17 (Fig. 7). The interaction was caused by the fact that color produced a larger reduction in verification times for the picture cues (*M* = 110.90, *SE*= 14.12) than for text cues (*M* = 60.31, *SE* = 15.30). This same interaction was observed in Experiment 1; however, in Experiment 1 adding color to a text-based cue had no impact on verification times, while here adding color resulted in faster verification times for both the picture-, *t*(40) = 7.85, *p* < .001, *d* = 1.23, and the text-based cues, *t*(40) = 3.94, *p* < .001, *d* = .62.

## Discussion

The results from Experiment 2 generally replicated the conclusion from Experiment 1. Verification times were again much faster for picture- than text-based cues, supporting the hypothesis that a high-fidelity cue should be beneficial to the verification process because the target is at fixation, and thus its visual representation should also be high-fidelity. In addition, adding color to a picture cue, so that it perfectly matches the target, resulted in extremely fast verification times. However, unlike Experiment 1, here adding color to the text-based cue also reduced verification times. This verification benefit for text-based cues that included the precise color of the dominant color of the target may be due to the match of the color supporting rapid verification.

Experiment 2 again found evidence that color information plays a major role in search guidance. For all dependent variables associated with the efficiency of search guidance (scanning time, rapid guidance, and the number of fixated items), text-based cues that include color information outperformed picture-based cues devoid of color. These findings are consistent with the hypothesis that much of the previously reported guidance benefit of picture-based over text-based cues may be due to the inclusion of color information in the former.

However, it is worth noting that even though Experiment 2 presented the color word of the cue in the dominant color of the target, adding that level of specificity to the color information provided little benefit above simply presenting the color word in grayscale. As a result, the color picture cue was more beneficial than a text-based cue with color information across all dependent variables. In short, although color information plays a large role in guidance, form information also plays a role.

One aspect did not replicate between experiments. In Experiment 1 adding color information to a cue (text- or picture-based) resulted in shorter distractor dwell times, suggesting that a mismatch between the cue’s color and a distractor helped one rapidly determine that the distractor was not the target. However, in Experiment 2 the inclusion of color information did not decrease distractor dwell times for either cue format. This failure to replicate raises concerns about concluding that color mismatch information has a robust impact on the process of identifying an item as a distractor.

It is worth noting that, unlike Experiment 1, in Experiment 2 the accuracy data presents some challenges for interpretation. In the grayscale conditions there appears to be a speed-accuracy tradeoff, with the faster hit RTs for the grayscale picture condition being coupled with lower accuracy for that condition. While this type of speed-accuracy tradeoff is not ideal, we are not as worried about this pattern as we would be if we did not have eye-tracking data to help interpret these data. That is, we are not relying heavily on search RTs to infer search guidance, but instead have a number of additional measures of search behavior that provide more direct measures of search guidance. Across all of these metrics (faster first fixations, faster distractor identification times, fewer items fixated during a target-present trial, and more rapid guidance to the target), the gray-scaled picture cue shows superior guidance to the text cue without color information.^4^ In short, these consistent search metrics and their relatively consistent pattern across both experiments give us confidence in our interpretations, despite the possible speed-accuracy tradeoff.

Finally, there were relatively high false alarm rates with text-based cues in both experiments. While again this is not ideal, it does not radically alter our interpretation of eye-tracking based search metrics. It is worth noting that while the false alarm rates in these conditions seem high, similarly high false alarm rates were found by Maxfield and Zelinsky, who found false alarm rates of 9.38% (*SE* = 2.1) for subordinate cues – cues that are similar to the ones we used here. Why these types of cues produce high false alarms is unclear, but do not fundamentally alter our interpretation of search guidance and verification time data.

## General discussion

We set out to re-examine the effect of high-fidelity search cues on search guidance and verification times. Our initial supposition was that prior work (Malcolm & Henderson, 2010; Maxfield & Zelinsky, 2012) showing a benefit to both guidance and verification times of picture over text-based cues confounded two factors – the picture cues included additional information about both color and high-fidelity form information. Based on prior research by Williams (1967), we reasoned that the additional color information might be responsible for the guidance benefit, while the high-fidelity form information might be responsible for the verification time benefit. To examine this issue, we included four cues that were the factorial combination of a 2 (color/grayscale) × 2 (picture/text) design. Doing so allowed us to independently evaluate the role of the high-fidelity form information provided by a picture as well as the role of color information.

Our results suggest that color information in a cue is a major factor in efficient guidance and more impactful than high-fidelity form information. Across a number of search guidance metrics, a text-based cue with color information was more effective than a gray-scaled picture cue. While color information alone could account for much of the search guidance afforded by a cue, it was not responsible for all of the guidance; a colored picture cue produced better guidance than a text-cue with color information, even when a color word in a text-based cue was presented in the precise color of the target’s dominant color. This finding suggests that providing high-fidelity form information produces a small but reliable benefit to search guidance above and beyond color information.

By contrast, for verification times high-fidelity form information seems to be more important than color information as gray-scaled picture cues produced faster verification times than text-based cues with color information. In fact, in Experiment 1 adding an abstract color name to a text-based cue did not significantly improve verification times, suggesting there is little role for color information in the verification process. However, when precise color information was presented in the cue, as in Experiment 2, that precise visual information did support faster verification times. Thus, for color information to benefit target verification times, the color information must be presented visually rather than as a conceptual color label.

In short, for verification times high-fidelity form information has a large impact while abstract color information has little impact. During the verification process the target is at, or very near, fixation, and thus the visual representation of the target should be a fairly high-fidelity representation that closely matches the high-fidelity cue represented in working memory. This close match between the fidelity of representations supports fast verification times.

By contrast, while form information presented in a picture-based cue has some influence on search guidance, color information, even that provided by an abstract color label, has a major impact on search guidance. Search guidance involves efficiently selecting which object to fixate next, based on visual representations of objects not directly at fixation. Given the rapid decrease in visual acuity with eccentricity (Staugaard et al., 2016), including high-fidelity form information in a cue should have little impact on guidance since the visual representation of peripheral objects should not include the high spatial frequencies necessary for a high-fidelity representation (Larson & Loschky, 2009). Color information, however, is well represented within the low spatial frequencies available for peripheral objects (Cajar et al., 2020), making it a good cue for guidance.

There may also be a second reason why color is such a strong factor for attentional guidance as there are fairly direct connections between higher area representations of color and lower-level perceptual units coding for that item. To elucidate this idea, consider how cues or attentional templates are thought to influence attentional selection (see Woodman et al., 2013, for review). Both theoretical (Desimone & Duncan, 1995) and computational (Bundesen et al., 2005) models suggest that search templates are represented in working memory at a somewhat late location in the processing stage, such as the inferotemporal cortex (Chelazzi et al., 1998). This representation is thought to bias attentional selection toward items in the environment that match the search template by back-propagating activity to earlier perceptual neurons responsible for coding the features associated with the target. This back-propagation makes these perceptual units more sensitive to in-coming visual stimuli that match the target, thereby giving them a competitive advantage for attention.

Based on this type of model, the efficiency of guidance will depend on how well the representation held in late working memory regions can activate early perceptual neurons. In this respect, color is particularly unique because color can be well represented at late working memory areas (Allred & Flombaum, 2014), and there are fairly specific low-level units associated with coding color throughout the visual processing stream (Seymour et al., 2009) and going as far forward as the retina (Conway, 2009). Thus, the activation of a given color in working memory may have a relatively well-defined and finite set of early units to target during the back-propagation process. By contrast, form information may have a far less well-defined and broader set of possible low-level units associated with that process form. This is because complex form information must be accumulated from the combination of several lower-level feature units (Treisman, 1996) and because the specific low-level units associated with coding a given form may vary depending on the viewpoint of that object (Kinchla & Wolfe, 1979).

Before closing, it is worth noting an alternative interpretation of these findings. Namely it is possible that the types of cues we compared require different amounts of time to become effective and that those differences in time to effectiveness might be responsible for our observed effects. For instance, suppose it takes longer for one to activate a search template when given a text-based cue than a picture based-cue. While we cannot definitively rule out this alternative, we think it highly unlikely given prior work by Wolfe and colleagues (Wolfe et al., 2004). In that work, the authors varied the time between the onset of the cue and search array to determine the time required for cues to reach their maximal guidance. They did this for a variety of cue types including picture cues, word cues, and categorical word cues. While they did find that specific picture cues produced better overall guidance and required less time to reach their maximal benefit, even their text-based categorical cues reached maximal benefit by 800 ms. By comparison, in our experiments the cues were available for a full 1,400 ms prior to the onset of the array, which should have provided ample time for even the text-based cues to reach their maximal benefit.

## Conclusions

We investigated the independent contribution of form and color information in a search cue on search guidance and verification times. Our results show that color is important for efficient guidance but does not completely account for the highly efficient guidance afforded by a colored picture-based cue as the form information provides additional benefit to guidance above color. By contrast, form information provided by a picture cue is important for efficient verifications times, with color playing less of a role. These results suggest that different features of a cue differentially impact different aspects of the search process, and suggest that designs interested in search processes should consider each source of information independently.

## Footnotes

1 We note that Malcom and Henderson found very large effect sizes (~ 1.02) for their comparison of search guidance for picture verse text-based cues. However, they used real-world scenes and did not separate out the effects of color and form, thus we thought it more appropriate to use a much smaller anticipated effect size.

2 2The Hemera Photo Objects II is a set of 50,000 royalty free images of single objects isolated from their background. We provide both the color and gray-scaled versions of the stimuli we used on OSF (10.17605/OSF.IO/5PU2S).

3 Malcolm and Henderson separated the search initiation time from the scanning time, so their scanning metric subtracted the time to first saccade initiation from the scanning time, but across all three experiments they found that cue type did not affect the saccade initiation time. Therefore, we have not eliminated that time from our scanning time, making our metric more similar to Maxfield and Zelinsky’s.

## Declarations

### Open Practices Statement

The images we used and SPSS formatted data for both experiments is available here https:// doi. org/https:// doi. org/ 10. 17605/ OSF. IO/ 5PU2S. Neither experiment was pre-registered.
